# Improved Mechanical Properties and Energy Absorption of BCC Lattice Structures with Triply Periodic Minimal Surfaces Fabricated by SLM

**DOI:** 10.3390/ma11122411

**Published:** 2018-11-29

**Authors:** Miao Zhao, Fei Liu, Guang Fu, David Z. Zhang, Tao Zhang, Hailun Zhou

**Affiliations:** 1 State Key Laboratory of Mechanical Transmission, Chongqing University, Chongqing 400044, China; zhaomiaocqu@gmail.com (M.Z.); liufei29@cqu.edu.cn (F.L.); guangfu@cqu.edu.cn (G.F.); zghzt@hotmail.com (T.Z.); Allenzhou@cqu.edu.cn (H.Z.); 2 College of Engineering, Mathematics and Physical Sciences, University of Exeter, North Park Road, Exeter EX4 4QF, UK

**Keywords:** lattice structures, mechanical properties, energy absorbing, triply periodic minimal surface, selective laser melting

## Abstract

The triply periodic minimal surface (TPMS) method is a novel approach for lattice design in a range of fields, such as impact protection and structural lightweighting. In this paper, we used the TPMS formula to rapidly and accurately generate the most common lattice structure, named the body centered cubic (BCC) structure, with certain volume fractions. TPMS-based and computer aided design (CAD) based BCC lattice structures with volume fractions in the range of 10–30% were fabricated by selective laser melting (SLM) technology with Ti–6Al–4V and subjected to compressive tests. The results demonstrated that local geometric features changed the volume and stress distributions, revealing that the TPMS-based samples were superior to the CAD-based ones, with elastic modulus, yield strength and compression strength increasing in the ranges of 18.9–42.2%, 19.2–29.5%, and 2–36.6%, respectively. The failure mechanism of the TPMS-based samples with a high volume fraction changed to brittle failure observed by scanning electron microscope (SEM), as their struts were more affected by the axial force and fractured on struts. It was also found that the TPMS-based samples have a favorable capacity to absorb energy, particularly with a 30% volume fraction, the energy absorbed up to 50% strain was approximately three times higher than that of the CAD-based sample with an equal volume fraction. Furthermore, the theoretic Gibson–Ashby mode was established in order to predict and design the mechanical properties of the lattice structures. In summary, these results can be used to rapidly create BCC lattice structures with superior compressive properties for engineering applications.

## 1. Introduction

Lattice structures, as multifunctional materials, provide a relatively low density accompanied by high strength, energy absorption, and heat conduction capabilities, which have potential applications in the aerospace, automobile, and biomedicine fields [1,2,3,4]. However, due to the micro unit size and complex internal configurations, conventional manufacturing techniques with high cost are unable to produce complex lattice structures, thus limiting lattice applications.

With the advent of additive manufacturing (AM) technology, parts with complex internal configurations, such as lattice structures, are now realizable. Selective laser melting (SLM) technology is a type of AM technology that builds up parts using a scanning laser beam to fuse successive layers of metal powders under an inert gas atmosphere. Compared to conventional fabrication approaches to produce metallic porous structures, such as melt gas injection [5], investment casting [6], physical vapor deposition [7], and the sheet metal technique [8], SLM technology has a variety of advantages, including efficient material utilization, time-saving capabilities, and manufacturing capabilities for complex structures. Therefore, this technology has been used to fabricate lattice structures in engineering applications [9,10,11,12]. This state-of-the-art manufacturing technology is giving designers greater freedom to design and optimize the geometry of parts without concerns regarding manufacturability [13].

Lattice structures comprising strut-like members can be classified as stretching-dominated and bending-dominated structures, according to the number of struts and nodes in their internal configurations through Maxwell’s stability criterion [14]. Stretching-dominated structures have higher stiffness and strength [15], while bending-dominated structures have a better specific energy absorption behavior for a long stress plateau under compression [16]. The body centered cubic (BCC) is a typical type of bending-dominated structures, and this structure has received great attention and been experimentally and theoretically analyzed for its special mechanical and energy absorbing performances [3,17,18,19,20]. For instance, the mechanical properties of the BCC lattice structure was experimentally investigated with different loading conditions [19] or loading directions [20], and the classical beam theory approach was used to predict its mechanical properties [17,18]. In addition, the drop weight impact tests demonstrated that a BCC lattice structure made of Ti–6Al–4V had a favorable performance in foreign object impact compared to aluminum honeycomb [3], and compression tests showed that a BCC lattice structure made of 316L appeared to be ideal for the design of impact absorption systems, owing to the long plateau-like low hardening regime prior to densification [20]. However, most of their designs were strut-like lattice structures generated by CAD-based commercial software in which the design and representation combined simple geometric objects through Boolean operations, which are more time-consuming for the designer and resource-consuming for the computer [21]. Furthermore, these strut-like lattice structures tend to fracture near the node due to stress concentration during loading, which in turn results in low mechanical properties for lattice [3,22].

Currently, a more efficient modeling method, named the triply periodic minimal surface (TPMS) method, has emerged as a novel tool for lattice design. The architectural features and volume fractions of lattice structures can be altered readily, and even graded lattice structures can be realized by modifying the mathematical formulas [23]. Melchels et al. [24] designed two well-known lattice structures called gyroid and diamond lattice using TPMS formulas, and a graded gyroid structure was generated by adding a linear *z*-value term to the formula. Similarly, primitive, I-WP, graded primitive, and graded diamond structures were designed using the same approach [25,26,27]. Moreover, Yang et al. [28] applied the sigmoid function and Gaussian radial basis function to connect two different types of TPMS-based lattice structures through smooth transition boundary cases, and Wolfram Mathematica software was used to directly generate an STL file for AM. Based on these functional defined lattice distributions in the spatial area, strengthening critical regions with a particular lattice in type or volume fraction becomes feasible and leads to optimized designs for engineering applications. In addition to the TPMS algorithm, the mechanical and biological properties of TPMS-based lattice structures have also been investigated [26,29,30,31,32]. The smooth convergence of struts can reduce stress concentrations [31], and a high surface to volume ratio can help cells to adhere and stimulate cell ingrowth in biomedical applications [32].

In order to rapidly create BCC lattice structures using the TPMS formula and fabricate them by SLM for engineering applications, the mechanical properties, failure mechanism, and energy absorption performance of TPMS-based BCC lattices must first be investigated in detail. It is worth highlighting that previous experimental studies and numerical analyses have found that these compressive properties of BCC lattice structures are significantly related to their local geometric features [17,18,33,34,35]. Ushijima et al. [17,18] theoretically studied BCC lattice structures using the classical beam theory approach, and the results indicated that the elastic modulus and yield strength were related to the diameter and the length of struts. By changing the diameter of the struts in a linear variation in the load direction, the failure mode of a BCC lattice changed to distinctive layer-by-layer deformation under quasi-static loading, resulting in an improvement in energy absorption performance [33]. Smith et al. [34] found that the load-bearing capability of BCC lattice structures can be enhanced by adding a vertical strut in each lattice unit. Owing to the extra vertical struts, the relationship between struts and nodes was changed, leading to the deformation of lattice structures being transformed from a bending-dominated to a stretching-dominated deformation [35]. Furthermore, the mechanical properties of lattice structures also can be improved by optimizing the area of the nodes and struts [20,22,36] or increasing the unit-cell size in the load direction [18].

Compared to current CAD-based lattice structures, TPMS-based ones exhibit distinctive local geometric features, with a continuous and smooth convergence of struts [27]. However, as an important factor influencing compressive properties (mechanical properties, failure mechanism, and energy absorption performance), the changed local features of TPMS-based BCC lattice structures have received little attention. Therefore, the aim of this study was to uncover how local geometric features of TPMS-based BCC lattice structures influence their compressive properties, and further to compare TPMS-based BCC lattice structures with CAD-based ones for their potential applications as protective and energy absorptive structures. Firstly, lattice structures with different volume fractions (from 10 to 30%) were designed using the TPMS formula, as well as the CAD-based method for comparison. Secondly, three duplicates of each lattice structure were fabricated using SLM, and then uniaxial compression experiments and SEM were performed to investigate their mechanical properties and failure mechanism. Thirdly, the theoretic Gibson–Ashby mode was established in order to predict the elastic modulus and yield strength of the lattice structures. Finally, the energy absorption of lattice structures was also investigated to assess their potential application for impact protection.

## 2. Materials and Methods

### 2.1. Design of BCC Lattice Structure

**Figure 1:**
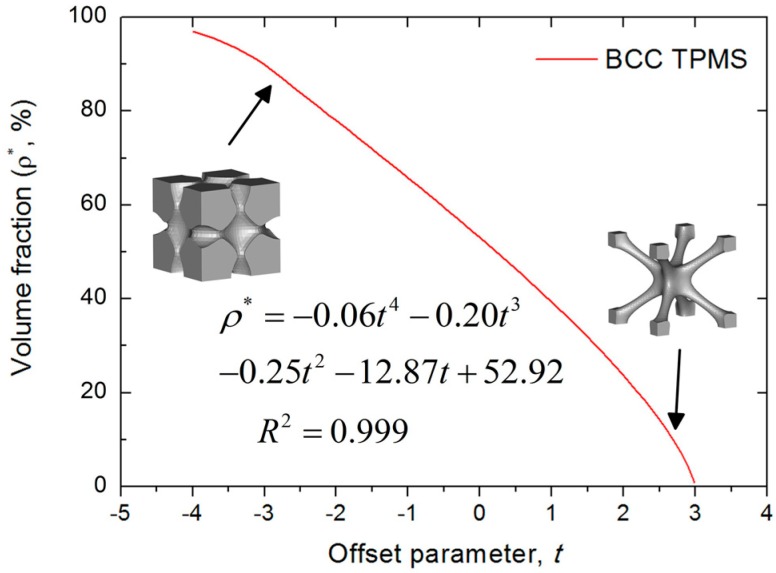
The relationship between volume fraction ($\rho^{*}$) and parameter t.

The TPMS algorithm and equations for the most common TPMS geometries were illustrated in Reference [37]. The equation describes the boundary between the void and solid material for lattice structures. For a BCC lattice structure, the boundary can be defined by the TPMS equation as follows:

(1) $$\begin{array}{c} F\left(x,y,z\right)=\cos\left(2k_{x}x\right)+\cos\left(2k_{y}y\right)+\cos\left(2k_{z}z\right)\\ -2\left(\cos\left(k_{x}x\right)\cos\left(k_{y}y\right)+\cos\left(k_{y}y\right)\cos\left(k_{z}z\right)+\cos\left(k_{z}z\right)\cos\left(k_{x}x\right)\right)+t=0 \end{array},$$

where t is an offset parameter used to control the position of the boundary between the void and solid material. $k_{i}\left(i=x,y,z\right)$ are the TPMS function periodicities used to control the unit size in the x, y, and z directions, written as:

(2) $$k_{i}=2\pi \frac{n_{i}}{L_{i}}\;\left(i=x,y,z\right),$$

where $n_{i}$ is the amounts of lattice unit in the x, y, and z directions; $L_{i}$ is the absolute sizes of $n_{i}$ units in x, y, and z directions; and the range of intervals in x, y, and z represents the external dimensions of the whole lattice structure. In order to represent BCC lattice structures with the TPMS method, the regions where F(x,y,z)≤0 are defined to be solid and the regions where F(x,y,z)>0 are defined to be void. Therefore, the volume fraction ($\rho^{*}$) (i.e., the proportion of solid regions in space for a lattice structure) of BCC lattice structures can be calculated by [38]:

(3) $$\rho^{*}=\frac{∭\left(F\left(x,y,z\right)\leq 0\right)\cap \left(\left|x\right|\leq L/2\right)\cap \left(\left|y\right|\leq W/2\right)\cap \left(\left|z\right|\leq H/2\right)dxdydz}{L\cdot W\cdot H},$$

where L, W, and H are the length, width, and height of the lattice structure, respectively. The position of the boundary between the void and solid material is varied by parameter t, which can generate larger or smaller solid regions to control the volume fraction ($\rho^{*}$) of the BCC lattice structure. In order to rapidly obtain BCC lattice structures with particular volume fractions, a nonlinear fitted relationship between parameter t and volume fraction ($\rho^{*}$) is shown in Figure 1, which is different from the linear relationship for gyroid lattice structures [24,25] and diamond lattice structures [25]. This novel approach allows designers to select parameter t to rapidly obtain a desired volume fraction for BCC lattice structures using the fitted equation $\rho^{*}=-0.06t^{4}-0.2t^{3}-0.25t^{2}-12.87t+52.92$.

**Figure 2:**
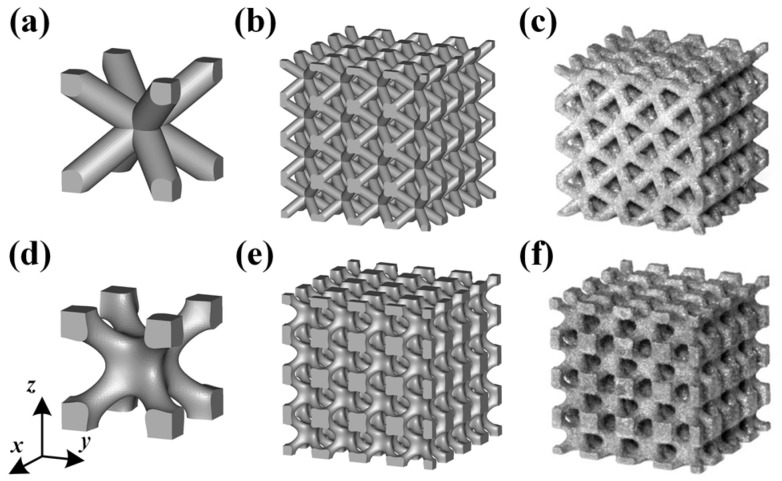
(**a**–**c**) Computer aided design (CAD) based body centered cubic (BCC) lattice structures and (**d**–**f**) triply periodic minimal surface (TPMS) based BCC lattice structures, from three-dimensional models to manufactured samples.

In this study, the external dimensions of lattice models were 16 mm × 16 mm × 16 mm, and each model contained a basic BCC unit cell with dimensions of 4 mm × 4 mm × 4 mm, resulting in a 4 × 4 × 4 lattice arrangement. The volume fractions of CAD-based and TPMS-based lattice structures were set at 10%, 20%, and 30% for comparison. As a result, the diameter of struts for the CAD-based lattices were generated to be 0.58, 0.85, and 1.08 mm, denoted as B10, B20, and B30, respectively. For the TPMS-based structures, these were achieved by setting parameters x,y,z∈[0,16], $k_{x}=k_{y}=k_{z}=0.5*\pi$ and t to be 1.63, 2.193, and 2.666 in Equation (1), denoted as BT10, BT20, and BT30, respectively. The CAD-based samples were designed by Solidworks software, saved as STL files and processed in Magics software (Materialise Inc., Leuven, Belgium), as shown in Figure 2a,b. The TPMS-based samples were performed by a MATLAB (MathWorks Inc., Massachusetts, United States) code to generate STL files and processed in Magics software, shown in Figure 2d,e.

### 2.2. Materials

**Figure 3:**
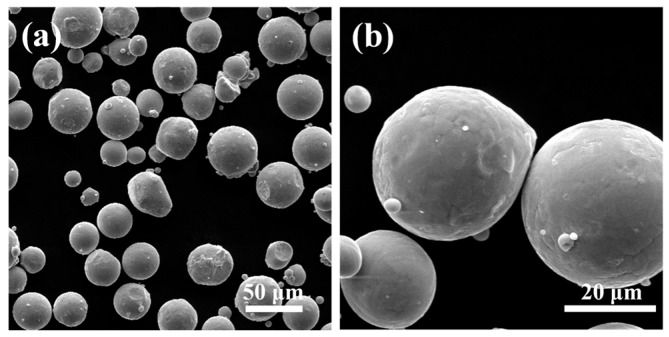
SEM micrographs of Ti–6Al–4V powder, (**a**) ×500 and (**b**) ×2000.

Scanning electron microscope (SEM) images of the commercial Ti–6Al–4V powder (Ti64-53/20, Tekna Advanced Materials Inc., Québec, Canada) used in this experiment are shown in Figure 3. The particle morphology exhibited a spherical shape with smooth surfaces, indicating a good flowability. The distribution of 93% particle size was in the range of 20–53 μm and the average particle size was 35.4 μm, which were measured by laser light diffraction (Malvern Panalytical Ltd., Malvern, UK).

### 2.3. Fabrication of BCC Lattice Structures

Three duplicates of each lattice structure and standard tensile specimen were fabricated through SLM technology using an EOSINT-M280 machine (EOS GmbH, Krailling, Germany). The available commercial processing parameters, including the heat treatment temperature from the EOS guidance document, were carried out to fabricate the experimental samples. The processing parameters were set up as follows: Laser power of 175 W, spot size of 0.1 mm, hatch spacing of 0.1 mm, layer thickness of 0.03 mm, scanning speed of 1250 mm/s, and $O_{2}$ content of less than 0.1%. The scanning strategy was an alternating hatch pattern where the direction of scanning was rotated by 67° between adjacent layers. Then, the lattice samples were removed from the substrate via wire electrical discharge machining (wire-EDM) (Suzhou Renguang CNC Equipment Co., Ltd., Suzhou, China) and subjected to heat treatment at 650 °C for 2 h with a furnace (Shanghai Jvjing Precision Instrument Manufacturing Co., Ltd., Shanghai, China) cooling step for relieving stress. Using these conditions, the density of bulk Ti–6Al–4V was measured as 4.42 g/mm^3^ (relative density ≥99%) through the Archimedes method.

### 2.4. Measurements

A universal mechanical testing machine (CMT5105, Shenzhen Wance Testing Machine Co., Ltd., Shenzhen, China) equipped with a 100 KN load cell was used for uniaxial compression testing with a strain rate of 1 mm/min, and three duplicates of each sample were subjected to uniaxial compression testing. Compressive force and displacement were recorded by a computer. In order to eliminate the influence of the build direction, the load direction was implemented parallel to the build direction (z direction). Two Canon 60D cameras (Canon Inc., Tokyo, Japan) together with appropriate lights were placed at the front and left side of the samples, and simultaneously recorded deformation of samples at a rate of 29 frames per second, providing information about the failure modes. The stress (σ) was obtained by dividing the compressive force by the apparent cross-sectional area of the lattice samples and the strain value (ε) was calculated by dividing the displacement by the initial height of the samples. Elastic modulus ($E_{L}$) was defined as the slope of its stress–strain curve in the elastic deformation region. Yield strength ($\sigma_{L}$) was defined as the stress at 0.2% plastic deformation. Moreover, the stress at the first peak on the stress–strain curve was regarded as the compression strength ($\sigma_{b}$).

A scanning electron microscope (SEM) (VEGA3 LMH, Tescan Inc., Brno, The Czech Republic) was used to characterize the morphology of the Ti–6Al–4V powder, the surface morphology of lattice samples before compression tests, and the fracture surfaces morphology after compression tests.

## 3. Results and Discussion

### 3.1. Geometry and Manufacturability

**Figure 4:**
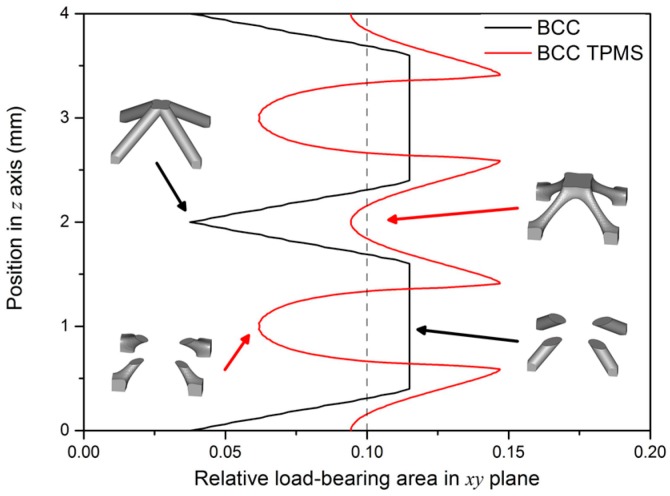
Relative load-bearing area of CAD-based and TPMS-based BCC unit cell along the load direction.

Although both lattice structures belong to the same BCC structure, there are still distinctions of geometry in local features, particularly for struts and nodes. CAD-based structures have uniform struts and small nodes with sudden transitions (Figure 2a), while TPMS-based structures have small struts and big nodes with smooth transitions (Figure 2d). Apart from that, the variation of the lattice load-bearing area in the xy plane for geometric evaluation is presented in Figure 4. From the figure, both lattices have lower relative load-bearing areas at nodes due to the overlapping of struts. However, for the TPMS-based lattice, in order to realize the smooth convergence of struts, the distribution of volume is transferred from centers of struts to nodes, leading to improvement in the node area, but weakening the strut area of the lattice.

**Figure 5:**
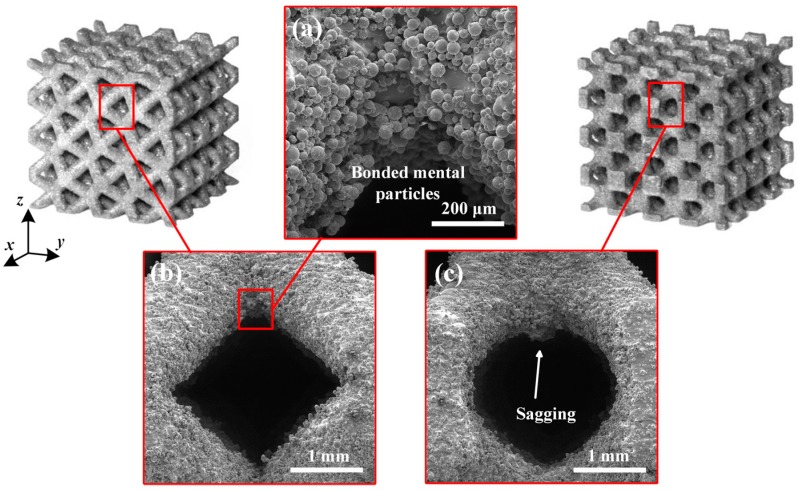
SEM micrographs of (**a**,**b**) CAD-based and (**c**) TPMS-based BCC lattice surfaces.

Both of the fabricated lattice samples are shown in Figure 2c–f, and their SEM micrographs are shown in Figure 5. The struts successfully converge on node and no broken cells are observed, which illustrates that these lattice structures can be successfully manufactured using SLM without the need for support structures. However, in Figure 5c, a slight sagging at the bottom of nodes for the TPMS-based lattice is observed. This is attributed to the smooth convergence of struts at the bottom of nodes where layers grow up rapidly, resulting in large changes in the area and position between two adjacent layers during SLM processing. As seen from a higher magnification SEM micrograph in Figure 5a, there exist a number of bonded spherical particles on the surfaces of lattice structures, and this phenomenon has also been found in other previous experimental studies [39,40]. It is worth noting that most bonded particles are in the range of 20–53 μm in particle size. Moreover, the particle morphology shows a smooth surface and a spherical shape in accordance with the bulk material in Figure 3, which indicates that the bonded particles are mainly caused by partially melted Ti–6Al–4V particles adhering to the surface of the part.

### 3.2. Deformation Behavior and Failure Mechanism

**Figure 6:**
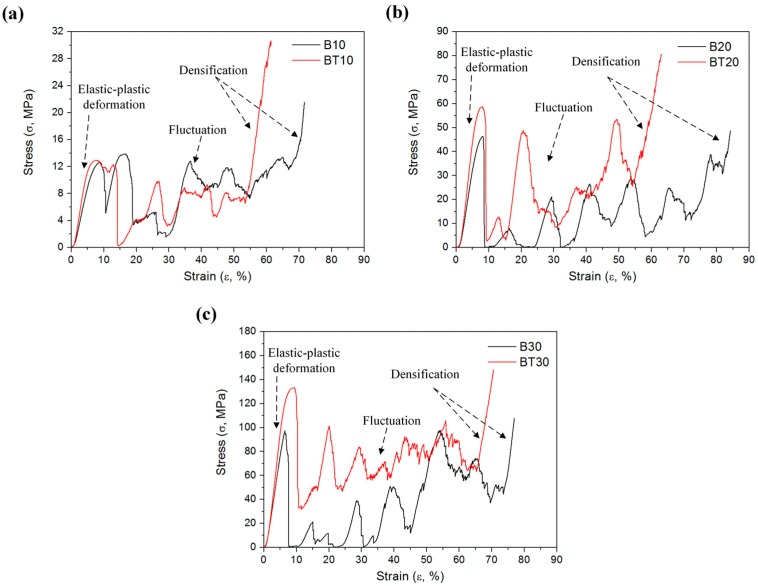
Stress–strain curves of CAD-based and TPMS-based samples in different volume fractions during compression tests. Volume fraction = (**a**) 10%, (**b**) 20%, (**c**) 30%.

**Figure 7:**
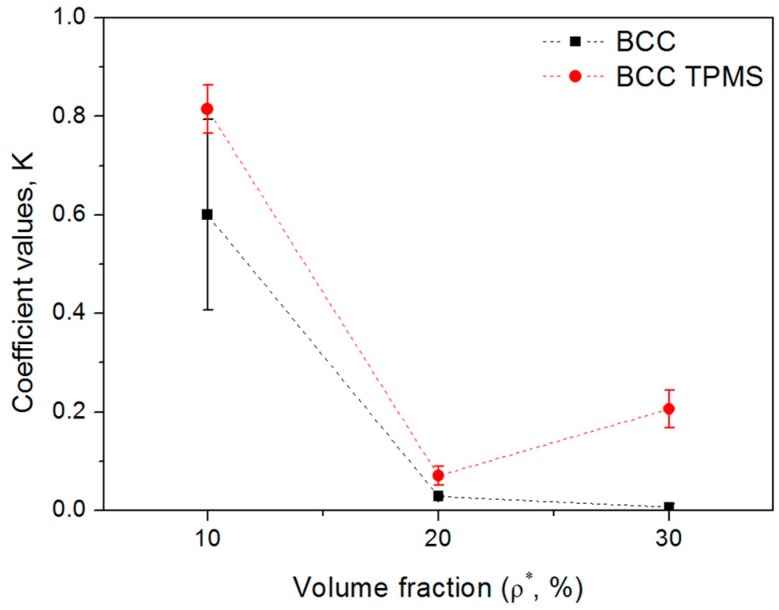
Load-bearing capability (K) of CAD-based and TPMS-based samples after the first plastic failure with different volume fractions (three replicate specimens, means and standard deviations).

The stress–strain curves of CAD-based and TPMS-based samples with different volume fractions during compression tests are illustrated in Figure 6, and their corresponding deformation behaviors are recorded in Table 1. As seen in Figure 6, there exist three distinct stages in terms of the elastic–plastic deformation stage, fluctuation stage, and densification stage, which is in accordance with the stress–strain curve of cellular metals in the ISO 13314:2011 standard [41]. Following the first stress peak, samples were cracked with a drop in the stress values due to the initial plastic failure, resulting in the low loading capacity of samples after plastic failure. These characteristics were also observed in aluminum–alloy porous structures [42,43] and Ti–6Al–4V lattice structures [44] in different designs and temperatures. In order to compare the load-bearing capability after the first plastic failure, coefficient K is carried out as follows:

(4) $$K=\frac{\sigma_{min}}{\sigma_{b}},$$

where $\sigma_{min}$ is the first lowest value of stress after the initial plastic failure and $\sigma_{b}$ is the compression strength, and regarded as the first stress peak. The maximum and minimum loading capabilities corresponded to 1 and 0, respectively. It can be seen from Figure 7 that the K values of the TPMS-based BCC samples were higher than those of the CAD-based ones, which indicates the TPMS-based structures have better load bearing capacity after being destroyed by compressive load. Moreover, the drop in the load-bearing capacity of lattice samples is affected by the volume fraction (i.e., larger volume fractions result in a smaller K). In a 10% volume fraction, lattice samples could retain most of their load-bearing capability (by 0.6 and 0.81 for B10 and BT10, respectively) and recover from initial abrupt collapse immediately. This was caused by the distinctive initial failure mode for B10 and BT10, in which the button layer collapses and the compressive load is rapidly and directly supported by the remaining part. By contrast, others exhibited a diagonal shear failure band, which led to two neighboring halves separating and sliding with weak load-bearing capability (less than 0.2). Similar results were observed in CAD-based cubic and honeycomb lattice structures by Choy [44] and TPMS-based gyroid lattice structures by Yang [45]. When the volume fraction increases, samples appear to behave like a solid block under loading conditions, and the resolved shear stress is at a maximum at 45° to the load direction [44]. The comparison in Figure 7 indicates that the loading-bearing capacity after the first plastic failure is determined not only by the types [44], loading temperature [43,46], and loading direction [46], but also by the volume fraction and local geometrical features.

**Figure 8:**
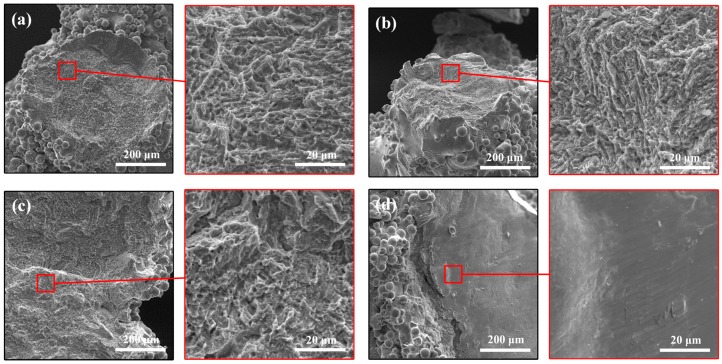
SEM micrographs of fracture surface of (**a**) B10, (**b**) BT10, (**c**) B30, and (**d**) BT30 after compression tests.

**Figure 9:**
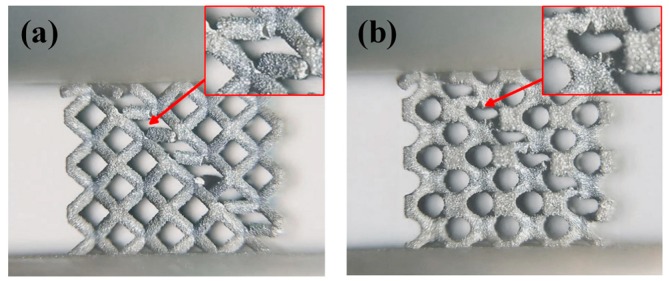
The fracture area of (**a**) CAD-based BCC lattice structures and (**b**) TPMS-based BCC lattice structures.

In order to further investigate details of their failure mechanism, fracture surfaces of lattice struts after the compression tests were observed by SEM and are shown in Figure 8. It can be seen that the fracture surfaces show different patterns from deep ductile dimples (Figure 8a–c) to smooth features (Figure 8d). For a lattice with a 10% volume fraction, the fracture surfaces of both types of samples were similar and deep ductile dimples were observed. These ductile fracture surfaces are found in the layer collapse mode of cubic and diamond lattice structures with a low volume fraction [22,44]. When the failure mode changed to 45° shear cracking in samples with a 30% volume fraction, B30 was found to exhibit a ductile fracture, while BT30 was found to exhibit a brittle fracture. The brittleness behavior corresponds to the strain–stress curve for BT30, showing higher frequency fluctuations (Figure 6c). Apart from that, previous experimental studies have found that lattice structures that fracture near nodes tend to exhibit ductile fracture [3,40]. This can likely be attributed to the strut area (where the scanned area was smaller than that of the nodes) containing a $\alpha^{\prime }/\alpha$ phase as a result of a higher cooling rate and subsequently showing signs of brittle fracture, while some sections close to the connecting nodes probably contain a β and subsequently show signs of ductile fracture [47]. It can be observed from Figure 9 that the fractures of the CAD-based BCC lattice structures are nearer to the nodes than those of the TPMS-based ones. This is basically attributed to the changed structural design, which is one important factor affecting the failure of lattice structures [48]. Therefore, it is worth analyzing the structures and stress distribution for these two types of lattices.

**Figure 10:**
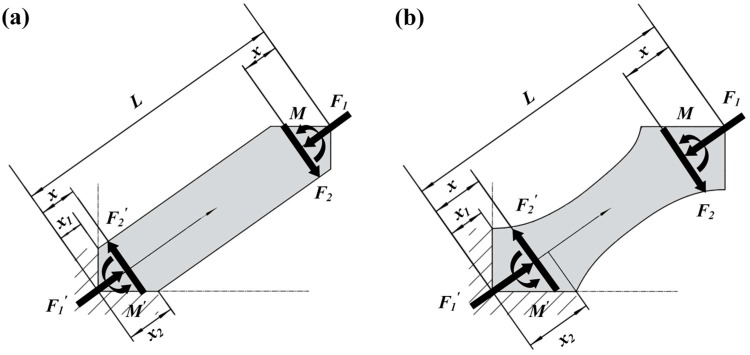
Simplified theoretic mode of (**a**) CAD-based BCC structures and (**b**) TPMS-based BCC structures.

According to the BCC lattice theoretic model by Gümrük [49], the reaction forces and moment act at the point offset a distance from the center of the joint due to the node effects, as seen in Figure 10, and this distance is assumed to be:

(5) $$x=\left(x_{1}+x_{2}\right)/2,$$

where $x_{1}$ and $x_{2}$ are defined as the distance from the center of the joint related to the node shape, and the maximum bending moment can be obtained as:

(6) $$M=F_{2}\left(L-2x\right)/2,$$

where $F_{2}$ is the shear force and L is the length of the strut, which is constant in this study. It can be observed from Figure 10 that the values of x vary depending on the local geometric features of the node and volume fractions. On the one hand, for TPMS-based BCC lattice structures, the values of x are higher than those of CAD-based ones. On the other hand, BCC lattice structures with higher volume fractions have higher values of x. Therefore, the bending moment of the BT30 sample becomes lower, which leads to the struts of BT30 being more affected by the axial force and struts with smooth features being subject to fracture. Meanwhile, struts of the B30 sample mainly undergo bending and fracture on nodes with ductile features. The results clearly demonstrate that even with the same type of lattice structure and failure mode, the failure mechanism can be totally changed by the local geometric features and stress distribution of the sample.

### 3.3. Mechanical Properties

It is known that the mechanical properties of lattice structures are affected by volume fractions, and the theoretic Gibson–Ashby mode was used to illustrate the relationship between mechanical properties and volume fraction [50]. For open-celled porous structures, these relations can be represented as follows:

(7) $$\frac{E_{L}}{E_{S}}=C_{1}{\left(\frac{\rho_{L}}{\rho_{S}}\right)}^{n_{1}},$$

(8) $$\frac{\sigma_{L}}{\sigma_{S}}=C_{2}{\left(\frac{\rho_{L}}{\rho_{S}}\right)}^{n_{2}},$$

where $E_{S}$, $\rho_{S}$, and $\sigma_{S}$ are the elastic modulus, density, and yield strength of the fully dense solid material, respectively; and $E_{L}$, $\rho_{L}$, and $\sigma_{L}$ are the elastic modulus, density, and yield strength of lattice structures, respectively. Constant parameters $C_{1}$, $C_{2}$, $n_{1}$, and $n_{2}$ are calculated by fitting the compression tests’ results. The properties of the solid material, $E_{S}=114.8\pm 32.5\;\mathrm{GPa}$, $\sigma_{S}=1029\pm 38.7\;\mathrm{MPa}$, are obtained by tensile test with standard tensile specimens according to the ISO 6892-1:2009 standard [51].

**Figure 11:**
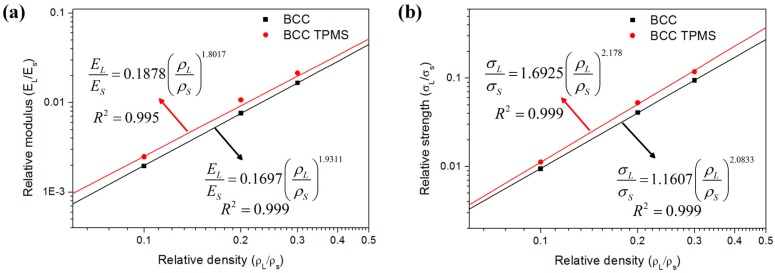
Variation of (**a**) the relative modulus and (**b**) the relative strength of the CAD-based and TPMS-based BCC lattice structures with relative density (three replicate specimens, means and standard deviations).

The mechanical properties of lattice structures from compression tests for all samples are summarized in Table 2. Figure 11a,b depicts log–log plots of the relative modulus against relative density values and the relative strength versus relative density values, respectively. These relative mechanical properties and relative density data points are fitted by Equations (7) and (8), and the fitted results with the coefficients of determination ($R^{2}$) are shown in Figure 11. Owing to these established relationships, the mechanical properties of lattice structures can be predicted. Moreover, lattice structures with certain mechanical properties can be achieved by adjusting the volume fraction.

As demonstrated in Figure 11, the fitted lines of TPMS-based structures dominate those of CAD-based ones, which illustrates that TPMS-based BCC lattice structures provide superior mechanical properties for protective applications. For TPMS-based samples with different volume fractions (10%, 20%, and 30%), the elastic modulus increased by 18.9%, 42.2%, and 26.4%, respectively; the yield strength increased by 19.2%, 29.5%, and 24.6%, respectively; and the compression strength increased by 2%, 26.2%, and 36.6%, respectively. This could be attributed to the following reasons. Firstly, the smooth convergence of struts in TPMS-based structures reduces stress concentration and enhances the load-bearing capability. Secondly, the distribution of volume for TPMS-based structures is transferred from the centers of struts to the nodes (Figure 4), resulting in an improvement in the node area, which is the weakest area of BCC lattice structures. Thirdly, the different local geometric features in design change the stress distribution (Figure 10) and cause TPMS-based samples to be more affected by axial force, while CAD-based samples are more influenced by the bending moment, resulting in the superior mechanical properties of TPMS-based samples. It should be noted that this change of stress distribution on struts is further confirmed by the fitted value of exponent $n_{1}$ for TPMS-based samples, which is lower than that of CAD-based ones, as shown in Figure 11a. This also indicates that CAD-based samples exhibit greater bending-dominated behavior than TPMS-based ones.

### 3.4. Energy Absorption under Compressive Deformation

**Figure 12:**
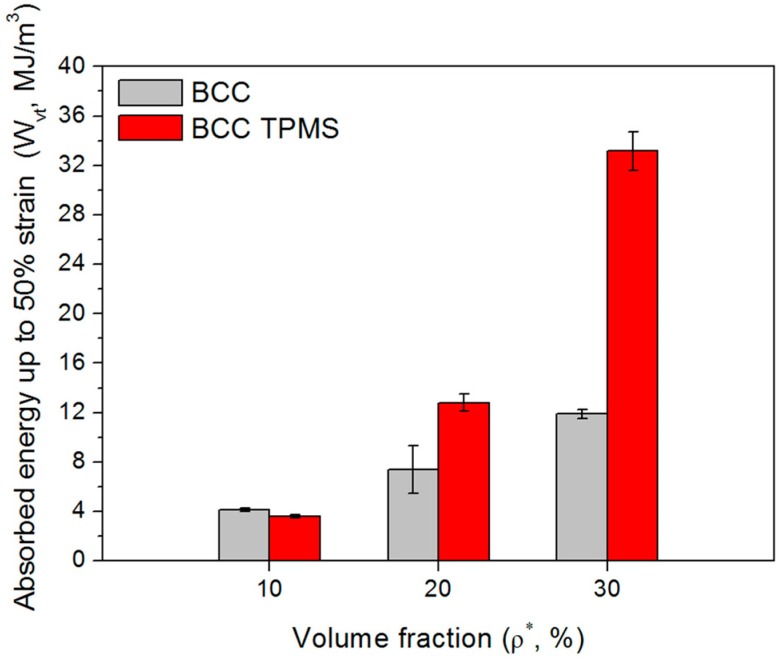
Comparison of energy absorbed per unit volume up to 50% strain, $W_{vt}$, for CAD-based and TPMS-based samples (three replicate specimens, means and standard deviations).

The energy absorption per unit volume up to 50% strain was calculated according to ISO 13314:2011 standard using the following equation [41]:

(9) $$W_{v}=\int_{0}^{0.5}\sigma \left(\varepsilon \right)d\varepsilon ,$$

where $W_{v}$ is the cumulative energy absorption per unit volume for lattice structures, ε is the strain, and σ(ε) is the stress related to ε during the compression tests. The values of $W_{v}$ of samples with 10% to 30% volume fractions were calculated by numerically integrating the compressive stress–strain curve, and are summarized in Table 3 and Figure 12. It can be observed that with the increase of the volume fraction, the energy absorption ability of CAD-based and TPMS-based BCC lattice structures is improved and the gap between these two types of lattices becomes larger. Particularly for BT30, the cumulative energy absorption per unit volume reaches 33.16 MJ/m^3^, which is three times higher than that of B30. Another method to enhance the energy absorption ability for BCC lattice structures is to design graded BCC lattice structures. However, compared to a uniform CAD-based BCC lattice with a 22% volume fraction, the graded one with an equal volume fraction only increased the value of total energy absorption by 10.5% [33].

**Figure 13:**
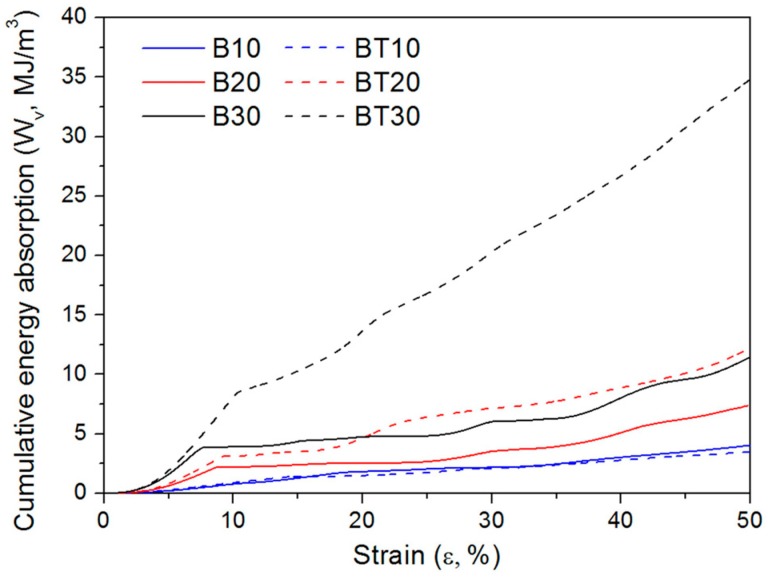
Curves between cumulative energy absorbing per unit volume ($W_{v}$) and strain (ε) of CAD-based and TPMS-based samples under compression test.

The cumulative values of calculated energy absorption per unit volume versus strain curves for samples under compression tests are demonstrated in Figure 13. It can be seen that the energy absorbing processes of these two types of lattices are similar. Initially, the rate of the cumulative energy absorption increases steadily, which is then followed by a decrease in the rate of the cumulative energy absorption due to the initial collapse of the lattice structures. Similar behavior has been found in BCC lattice structures with Al–Si10–Mg [33], as well as in cubic lattice structures and honeycomb lattice structures with Ti–6Al–4V [39].

**Figure 14:**
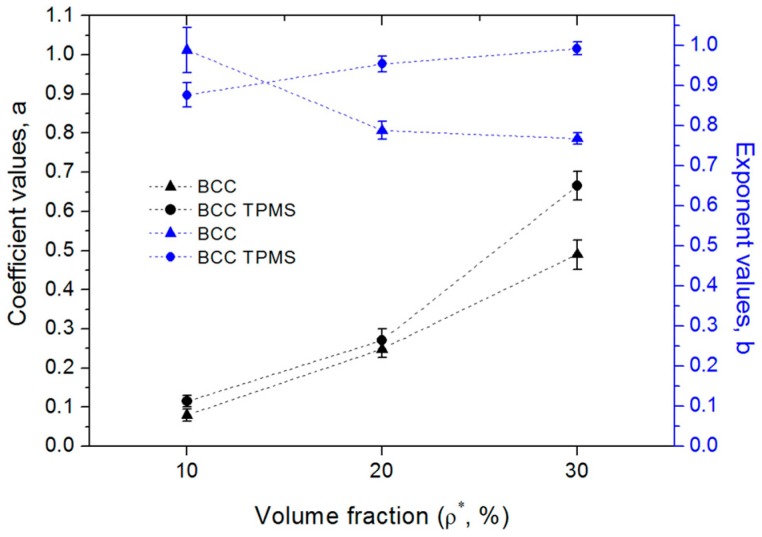
The fitted resulted coefficient values (a) and exponent values (b) of energy absorption curves (three replicate specimens, means and standard deviations).

In order to further investigate the energy absorption behavior of lattice structures, changes of $W_{v}$ with an increase of ε for all designs were fitted by power law and are tabulated in Table 4. The coefficient values (a) relate to the initial energy absorption performance, and the exponent values (b) represent the increasing rate of cumulative energy absorption of lattice structures under compressive loads. As demonstrated in Figure 14, the coefficient values (a) of all samples increase with the volume fraction, and the TPMS-based samples exhibit higher values of a than those of the CAD-based samples. This is principally because the TPMS-based samples or samples with a high volume fraction provide better resistance to deformation (high elastic modulus), meaning that these samples absorb more energy in the initial period. For CAD-based BCC lattice structures, the exponent value (b) of B10 is around 1 and exhibits a nearly linear relationship with strain values, while the rate ranges of B20 and B30 are both lower than 0.8. The lower exponent values of B20 and B30 could be attributed to their sudden 45° shear failure and low relative stress values in the fluctuating stage, leading them to exhibit a lower capacity in continuous energy absorption. However, the exponent value found by Maskery et al. [33] was around 1 for a CAD-based BCC lattice structure with a 22% volume fraction, being much higher than those of B20 and B30. This is because the material for that lattice structure was Al–Si10–Mg with good ductility, which illustrated high values of stress in the fluctuating stage. Apart from the material, it should be noted that exponent values of BT20 and BT30 were 0.931 and 1.014, respectively, and these values surpassed those of B20 and B30. The results clearly demonstrate that the changed local geometric features of BCC lattice structures can also improve the energy absorption behavior.

## 4. Conclusions

In this paper, we used the TPMS formula to generate BCC lattice structures with volume fractions in the range of 10 to 30% and successfully fabricated them using SLM with Ti–6Al–4V, providing a novel approach for designers to rapidly generate BCC lattice structures with particular unit sizes and volume fractions. Compression tests, theoretic analysis of struts, and SEM results showed that the struts of the TPMS-based samples were more affected by the axial force and were subject to brittle fracture, while struts of the CAD-based samples were mainly deformed by the bending moment and cracked on nodes with ductile fracture. The distinctive volume and stress distributions of struts rendered the TPMS-based BCC lattice structures superior to CAD-based ones, as the elastic modulus, yield strength, and compression strength increased in the ranges of 18.9–42.2%, 19.2–29.5%, and 2–36.6%, respectively. The energy absorption performance was also investigated, and it was found to vary with volume fractions and designs. TPMS-based structures with a high volume fraction were able to absorb more energy in a better energy absorption manner. Correlations between the mechanical properties and volume fraction were established by the theoretic Gibson–Ashby mode, which can be used to predict and design the mechanical properties of TPMS-based BCC lattice structures.

These results uncovered the mechanical properties, failure mechanism, and energy absorption performance of TPMS-based BCC lattice structures and demonstrated that these structures were competitive with CAD-based ones for protective and energy-absorbing applications, such as cycle helmet and packaging materials. However, our work only investigated uniform TPMS-based BCC lattice structures under compressive load. In order to better apply these lattice structures to engineering applications, the distribution of the lattice with particular volume fractions should be associated with the stress condition of the parts (i.e., graded lattice structures). Thus, future work should aim to investigate the mechanical properties, failure mechanism, and energy absorption performance of graded TPMS-based BCC lattice structures under complex loading conditions. Apart from that, fatigue performance of TPMS-based BCC lattice structures should be also investigated, as this is of vital importance in selecting a lattice for engineering applications.

## Figures and Tables

**Table 1:** Deformation of CAD-based and TPMS-based samples at different strains during compression tests.

Num	Strain (ε, %)					
	CAD-Based BCC			TPMS-Based BCC		
	0%	10%	20%	0%	10%	20%
B10/ BT10	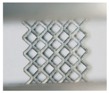	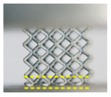	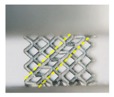	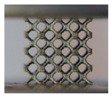	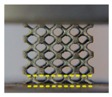	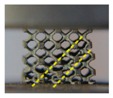
B20/ BT20	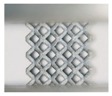	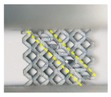	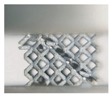	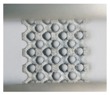	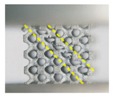	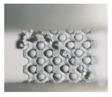
B30/ BT30	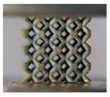	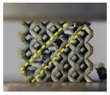	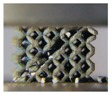	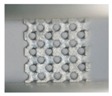	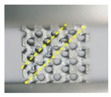	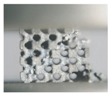

**Table 2:** Mechanical properties of CAD-based and TPMS-based samples, given as means ± standard deviations (three replicate specimens).

Num	Elastic Modulus $E_{L}\;(\mathrm{MPa})$	Yield Strength $\sigma_{L}\;(\mathrm{MPa})$	Compression Strength $\sigma_{b}\;(\mathrm{MPa})$
B10	224.6 ± 3.1	9.68 ± 0.32	12.87 ± 0.26
BT10	284.7 ± 13.9	11.55 ± 0.04	13.14 ± 0.24
B20	873.3 ± 15.4	41.97 ± 0.42	47.23 ± 0.85
BT20	1230.3 ± 10.1	54.25 ± 0.54	59.62 ± 0.94
B30	1904.4 ± 59.9	97.14 ± 0.57	98.77 ± 1.30
BT30	2451.1 ± 33.9	121.09 ± 1.79	134.93 ± 1.79

**Table 3:** The value of $W_{vt}$ for CAD-based and TPMS-based samples, given as means ± standard deviations (three replicate specimens).

	B10	BT10	B20	BT20	B30	BT30
$W_{vt}$ (MJ/m^3^)	4.13 ± 0.14	3.6 ± 0.13	7.39 ± 1.92	12.81 ± 0.68	11.88 ± 0.39	33.16 ± 1.57

**Table 4:** The fitted resulted equation for energy absorption curves, given as means ± standard deviations (three replicate specimens).

$W_{v}=a\varepsilon^{b}$	B10	BT10	B20	BT20	B30	BT30
a	0.085 ± 0.151	0.114 ± 0.015	0.248 ± 0.022	0.271 ± 0.029	0.490 ± 0.037	0.667 ± 0.037
b	0.989 ± 0.056	0.876 ± 0.027	0.788 ± 0.021	0.954 ± 0.020	0.769 ± 0.015	0.993 ± 0.016
